# Regulatory T Cell Suppressive Potency Dictates the Balance between Bacterial Proliferation and Clearance during Persistent *Salmonella* Infection

**DOI:** 10.1371/journal.ppat.1001043

**Published:** 2010-08-12

**Authors:** Tanner M. Johanns, James M. Ertelt, Jared H. Rowe, Sing Sing Way

**Affiliations:** Departments of Pediatrics and Microbiology, University of Minnesota School of Medicine, Center for Microbiology and Infectious Disease Translational Research, Minneapolis, Minnesota, United States of America; Stanford University School of Medicine, United States of America

## Abstract

The pathogenesis of persistent infection is dictated by the balance between opposing immune activation and suppression signals. Herein, virulent *Salmonella* was used to explore the role and potential importance of Foxp3-expressing regulatory T cells in dictating the natural progression of persistent bacterial infection. Two distinct phases of persistent *Salmonella* infection are identified. In the first 3–4 weeks after infection, progressively increasing bacterial burden was associated with delayed effector T cell activation. Reciprocally, at later time points after infection, reductions in bacterial burden were associated with robust effector T cell activation. Using *Foxp3* ^GFP^ reporter mice for *ex vivo* isolation of regulatory T cells, we demonstrate that the dichotomy in infection tempo between early and late time points is directly paralleled by drastic changes in Foxp3^+^ Treg suppressive potency. In complementary experiments using *Foxp3* ^DTR^ mice, the significance of these shifts in Treg suppressive potency on infection outcome was verified by enumerating the relative impacts of regulatory T cell ablation on bacterial burden and effector T cell activation at early and late time points during persistent *Salmonella* infection. Moreover, Treg expression of CTLA-4 directly paralleled changes in suppressive potency, and the relative effects of Treg ablation could be largely recapitulated by CTLA-4 *in vivo* blockade. Together, these results demonstrate that dynamic regulation of Treg suppressive potency dictates the course of persistent bacterial infection.

## Author Summary

The balance between immune activation and suppression is intricately controlled allowing optimal host defense against infection, while simultaneously minimizing collateral immune-mediated damage to host tissues. Although regulatory T cells have been implicated to play critical roles in sustaining this balance, their role in controlling the dynamic changes in immune cell activation during the natural progression of persistent infection are undefined. Herein, we explored the relative importance of regulatory T cells in controlling infection tempo using a model of persistent *Salmonella* infection representative of human typhoid. Early after infection when the bacterial burden is progressively increasing, the activation of protective immune components is delayed, and this coincides with increased regulatory T cell suppressive potency. Conversely, later during infection when reductions in bacterial burden occur, protective immune components are highly activated and regulatory T cell suppressive potency is markedly diminished. Moreover, the tempo of persistent *Salmonella* infection is controlled by regulatory T cells because ablation of these cells early after infection when their suppressive potency is increased accelerates bacterial eradication, while their ablation later when their suppressive potency is reduced causes no significant effects. Thus, regulatory T cell suppression controls the tempo of persistent *Salmonella* infection.

## Introduction

Typhoid fever is a systemic, persistent infection caused by highly adapted host-specific strains of *Salmonella* [1], [2], [3]. Human typhoid is caused predominantly by *S. enterica* serotype Typhi [4], while mice develop a typhoid-like disease following *S. enterica* serotype Typhimurium infection. Interestingly, the early stages of this infection, in both mice and humans, are usually asymptomatic or associated with only mild, non-specific “flu-like” symptoms [4], [5]. This represents a stark contrast to other Gram-negative bacterial pathogens (e.g. *Escherichia coli*, *Neisseria meningitidis*, *Haemophilus influenza*) that primarily cause acute infection and immediately trigger robust systemic symptoms after tissue invasion. Thus, the inflammatory response is blunted early after infection with *Salmonella* strains that cause persistent infection, and this feature likely facilitates long-term pathogen survival [3]. On the other hand, the blunted inflammatory response to systemic *Salmonella* infection also minimizes immune-mediated damage to host tissues that may outweigh the immediate risk posed by the pathogen itself [6]. Thus, dampening the immune response provides potential advantages to pathogen and host during persistent *Salmonella* infection.

Regulatory T cells (Tregs) were initially identified as a CD25-expressing subset of CD4^+^ T cells required for maintaining peripheral immune tolerance to self-antigen. However more recent studies clearly demonstrate their importance extends to controlling the immune response during infection [7], [8], [9], [10]. In this regard, the functional importance of Tregs has been best characterized for pathogens that cause persistent infection. For example, depletion of CD25^+^CD4^+^ Tregs is associated with enhanced effector T cell activation and reduced pathogen burden during *Leishmania major* infection [11]. Similarly, reconstituting T cell-deficient mice with CD25^+^CD4^+^ Tregs abrogates enhanced pathogen clearance that occurs after reconstitution with CD25-depleted CD4^+^ T cells [11], [12]. These complementary experimental approaches initially used to identify the role of CD25^+^ Tregs in host defense during *L. major* infection have since been reproduced after infection with numerous other bacterial, viral, and parasitic pathogens [8], [13], [14], [15], [16], [17], [18]. Interestingly, Treg-mediated immune suppression can also play “protective” roles for infections where host injury caused by the immune response outweighs the damage caused by the pathogen itself [13], [16], or when pathogen persistence is required for maintaining protection against secondary infection [11], [19]. Together, these findings suggest Treg-mediated immune suppression can provide both detrimental and protective roles in host defense against infection.

Despite these observations, identifying the functional importance of Tregs during *in vivo* infection has been limited, in part, by the lack of unique markers that allow their discrimination from other CD4^+^ T cell subsets. In this regard, the majority of infection studies have experimentally manipulated Tregs based on surrogate markers such as CD25 expression on CD4^+^ T cells. However, since CD25 expression is also a marker for activated T cells with no suppressive function, identifying Tregs based on CD25 expression does not allow discrimination between these functionally distinct T cell subsets. These limitations have been recently overcome by the identification of Foxp3 as the master regulator for Treg differentiation, and the generation of transgenic mice that allow precise identification or targeted manipulation of Tregs based on Foxp3 expression [20], [21], [22]. These include *Foxp3* ^GFP^ reporter mice that allow *ex vivo* Foxp3^+^ Treg isolation by sorting for GFP-expressing cells, and *Foxp3* ^DTR^ transgenic mice that co-express a high affinity diphtheria toxin receptor (DTR) with Foxp3 [23], [24]. Intriguingly, the first infection study using *Foxp3* ^DTR^ mice for Treg ablation revealed somewhat paradoxical roles for Foxp3^+^ Tregs in host defense. Within the first fours days after intravaginal herpes simplex virus 2 (HSV-2) infection, reduced inflammatory cell infiltrate and increased viral burden were found at the site of infection in Treg-ablated compared with Treg-sufficient mice [25]. These effects were not limited to HSV-2, nor were they restricted to the mucosal route of infection as increased pathogen burden associated with Foxp3^+^ Treg ablation also occurred after parenteral infection with lymphocytic choriomeningitis virus (LCMV) and West Nile virus [25], [26]. Whether these Treg-mediated reductions in pathogen burden are limited to these specific viral pathogens, or represent re-defined roles for Tregs based on their manipulation using Foxp3-specific reagents are currently undefined. Therefore, additional studies using representative mouse models of other human infections and Foxp3-specific reagents for Treg manipulation are required. In this study, the role of Foxp3^+^ Tregs in controlling immune cell activation and the balance between pathogen proliferation and clearance during the natural progression of persistent bacterial infection was examined after infection with virulent *Salmonella*.

## Results

### Persistent *Salmonella* infection in F1 129SvJ X C57BL/6 mice

**Figure 1:**
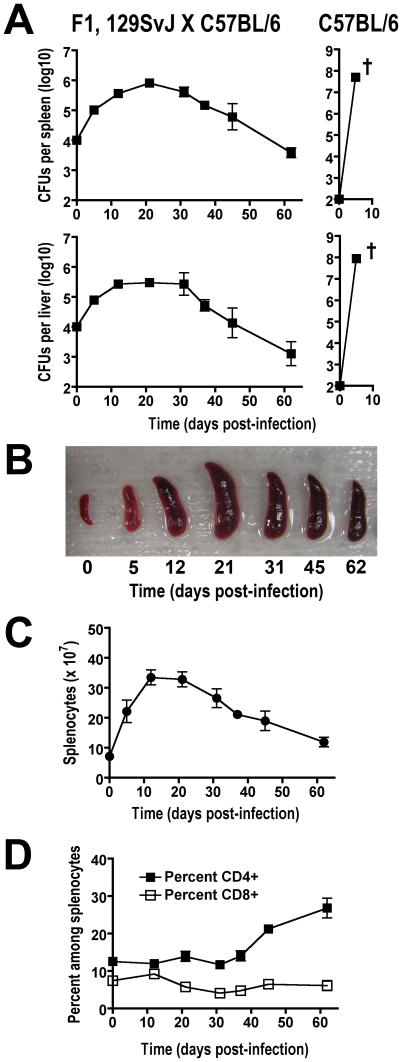
Tempo of persistent *Salmonella* infection in F1 129SvJ X C57BL/6 mice. A. Recoverable CFUs at the indicated time points after infection from the spleen (top) and liver (bottom) after infection with 10^4^ *S. enterica* serotype Typhimurium (strain SL1344) in F1 129SvJ X C57BL/6 (left) or 10^2^ in C57BL/6 (right) mice. †, all mice died or were moribund. B. Spleen size in F1 129SvJ X C57BL/6 mice at the indicated time points after infection. Absolute number of splenocyte cells (C) and percent CD4^+^ and CD8^+^ cells (D) in F1 129SvJ X C57BL/6 mice at the indicated time points after infection. These data reflect eight to ten mice per time point representative of three independent experiments. Bar, standard error.

Commonly used inbred mouse strains have discordant levels of innate resistance to virulent *S. enterica* serotype Typhimurium based primarily on whether a functional allele of *Nramp1* is expressed [27], [28]. For example, C57BL/6 mice express a functionally defective, naturally occurring variant of Nramp1 and thus, are inherently susceptible to infection with virulent *Salmonella* dying within the first few days from uncontrolled bacterial replication. By contrast, 129SvJ mice, which express wild-type Nramp1 (Nramp1-sufficient), are inherently more resistant developing a persistent infection instead [29], [30]. Since transgenic mouse tools for Treg manipulation based on Foxp3-expression are available primarily on the susceptible, Nramp1-defective C57BL/6 background, we sought to exploit the autosomal dominant resistance to *Salmonella* conferred by wild-type *Nramp1*, and the X-linked inheritance of *Foxp3* transgenic mice by examining infection in resistant F1 129SvJ X C57BL/6 mice [30]. Similar to results after infection with virulent *Salmonella* in 129SvJ mice, progressively increasing bacterial burdens are found throughout the first 3–4 weeks after infection in F1 129SvJ X C57BL/6 mice (Figure 1A). By contrast, Nramp1-defective C57BL/6 mice died within the first week after infection from overwhelming bacterial replication despite a 100-fold reduction in *Salmonella* inocula (Figure 1A). The progressively increasing bacterial burden within the first 3–4 weeks after *Salmonella* infection in F1 129SvJ X C57BL/6 mice parallels dramatic changes in both spleen size and absolute number of splenocytes (Figure 1B and C). Each of these parameters increased within the first three weeks after infection and declined subsequently at later time points that directly coincide with changes in *Salmonella* bacterial burden (Figure 1A–C). These findings demonstrate an interesting dichotomy in infection tempo between early (first 3–4 weeks) and later time points during persistent *Salmonella* infection in resistant F1 129SvJ X C57BL/6 mice.

### Delayed T cell activation early after *Salmonella* infection

**Figure 2:**
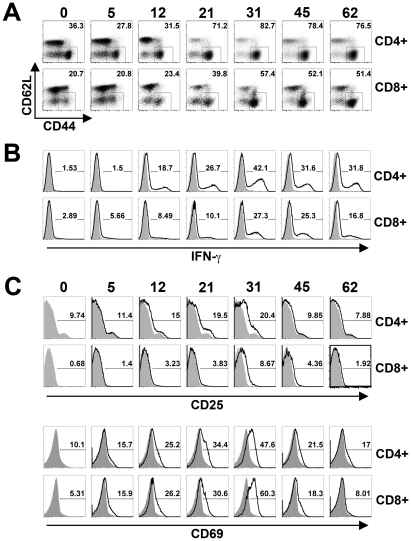
T cell activation kinetics during persistent *Salmonella* infection. A. Percent CD44^hi^CD62L^lo^ cells among CD4^+^ and CD8^+^ T cells at the indicated time points after infection with 10^4^ *S. enterica* serotype Typhimurium in F1 129SvJ X C57BL/6 mice. B. Percent IFN-γ producing CD4^+^ and CD8^+^ T cells after *Salmonella* infection and *ex vivo* stimulation with anti-CD3/CD28 antibody (black histogram) or no stimulation control (shaded histogram) at the indicated time points after infection. C. Expression levels of CD25 (top) and CD69 (bottom) by CD4^+^ and CD8^+^ T cells at the indicated time points post-infection (black histograms) compared to naïve F1 control mice (shaded histograms). These data reflect eight to ten mice per time point representative of three independent experiments.

Given the importance of T cells in host defense against *Salmonella* [31], [32], [33], the expansion and activation kinetics for CD4^+^ and CD8^+^ T cells during this persistent infection were each enumerated. Although the absolute numbers of both cell types increased in parallel with the absolute numbers of splenocytes, a progressive and steady increase in percent CD4^+^ T cells became readily apparent beginning week three post-infection (Figure 1D). By contrast, the percent CD8^+^ T cells remained essentially unchanged throughout these same time points. Additional phenotypic characterization revealed that the percent activated (CD44^hi^CD62L^lo^) CD4^+^ and CD8^+^ T cells both increased sharply beginning week 3, and were sustained at high levels through week 7 after infection (Figure 2A). Furthermore, the kinetics of T cell activation based on CD44 and CD62L expression directly paralleled the kinetics whereby CD4^+^ and CD8^+^ T cells each became primed for IFN-γ production (Figure 2B). Thus, the kinetics of CD44 and CD62L expression and IFN-γ production each reveal delayed T cell activation early after infection, not peaking until weeks 3 to 4, that is followed by more sustained T cell activation thereafter.

Given the durability whereby T cells maintain changes in CD44 and CD62L expression, and IFN-γ production after activation, the expression of more transient T cell activation markers such as CD25 and CD69 were also quantified throughout persistent *Salmonella* infection. CD25 and CD69 expression on CD4^+^ and CD8^+^ T cells each peaked between weeks 3 and 4 post-infection (Figure 2C). However consistent with the transient nature of their expression, CD25 and CD69 expression each declined to baseline levels over the next 2 to 3 weeks. Thus, the sharp increase in T cell activation that occurs between weeks 3 and 4 after *Salmonella* infection is confirmed using both transient (CD25, CD69) and more stable (CD44, CD62L, IFN-γ) markers of T cell activation. Interestingly, the overall kinetics for T cell activation beginning week 3 after infection directly parallels when reductions in bacterial burden begins to occur, and suggests dampened T cell activation early after infection allows progressively increasing bacterial burden, while enhanced T cell activation later facilitates bacterial clearance.

### CD4^+^ T cell-mediated *Salmonella* clearance during persistent infection

**Figure 3:**
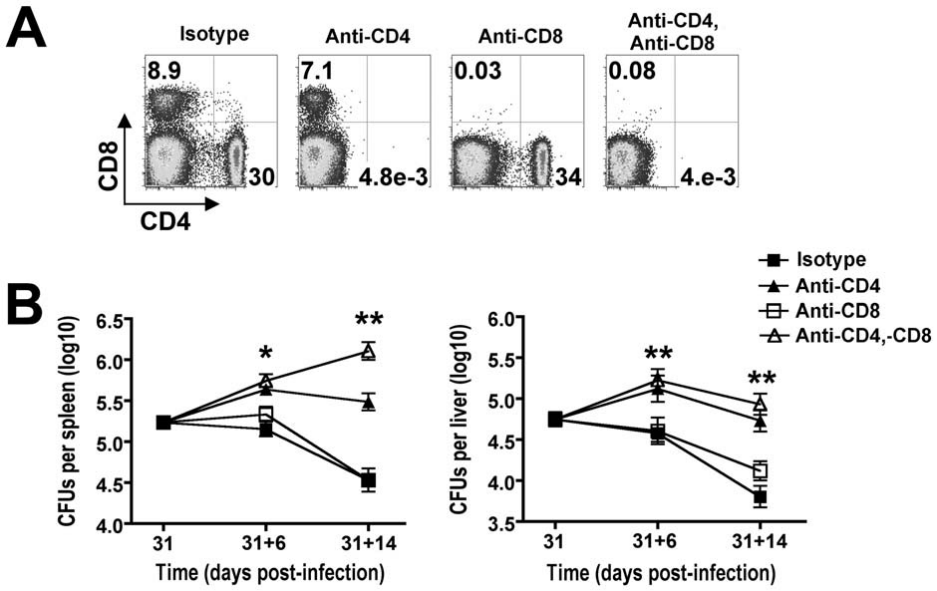
CD4^+^ T cells are required for reductions in *Salmonella* pathogen burden during persistent infection. A. Percent CD4^+^ and CD8^+^ T cells 14 days after treatment with each indicated antibody in mice beginning day 31 after *Salmonella* infection. B. Recoverable *Salmonella* CFUs in the spleen (left) and liver (right) for mice treated with each antibody for six days (31+6) or 14 days (31+14). These data reflect six to twelve mice per time point representative of three independent experiments each with similar results. Bar, standard error. *, *p*<0.05; **, *p*<0.001.

To determine the overall importance and individual contribution provided by each T cell subset in bacterial clearance during the natural course of persistent *Salmonella* infection, the impacts of CD4^+^ and/or CD8^+^ T cell depletion were determined. Anti-mouse CD4 and anti-mouse CD8 depleting antibodies were administered beginning day 31 post-infection. In initial studies, we found that 750 µg of each could deplete the respective T cell subset with ≥99% efficiency even in *Salmonella*-infected mice that contain expanded T cell numbers (Figure 3A). With sustained CD4^+^ T cell depletion, significantly increased numbers of recoverable *Salmonella* CFUs were found day 6 (day 31+6) after the administration of anti-mouse CD4 compared with isotype control antibody (Figure 3B). Moreover, the magnitude of this difference became even more pronounced by day 14 (day 31+14) after antibody treatment. By contrast, CD8^+^ T cell depletion alone or together with CD4^+^ T cell depletion did not cause significant changes in *Salmonella* bacterial burden except in the spleen day 14 after antibody treatment where combined depletion of both CD4^+^ and CD8^+^ T cells resulted in increased numbers of recoverable *Salmonella* CFUs compared to CD4^+^ T cell depletion alone (Figure 3B). Together, these results demonstrate an essential role for CD4^+^ T cells in the clearance of persistent *Salmonella* infection, and these findings are consistent with the previously reported requirement for this T cell subset in controlling the replication of attenuated *Salmonella* in susceptible Nramp1-defective mice [31]. Moreover, an essential role for CD4^+^ T cells in host defense during persistent infection in resistant mice is further supported by the sharp increase in overall percentage and activation of these cells which coincides with reductions in *Salmonella* bacterial burden beginning week 3 post-infection (Figure 1 and 2).

### Parallel expansion of Foxp3^+^ Tregs and non-Treg CD4^+^ cells during persistent infection

**Figure 4:**
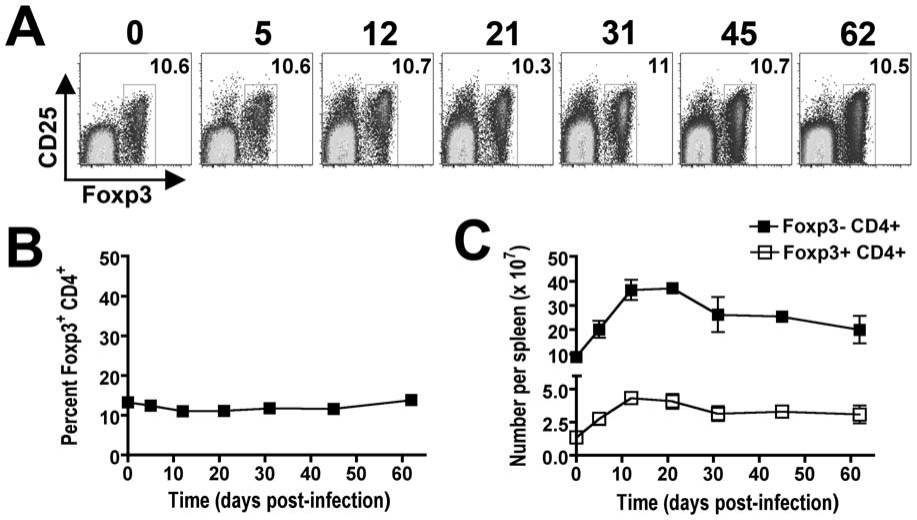
Parallel expansion of Foxp3^+^ and Foxp3-negative CD4^+^ T cells during persistent *Salmonella* infection. Representative FACS plots (A) and composite data (B) indicating percent Foxp3^+^ cells among CD4^+^ T cells at the indicated time points after infection with 10^4^ *Salmonella* in F1 129SvJ X C57BL/6 mice. C. Total numbers of Foxp3^+^CD4^+^ Tregs and Foxp3-negative non-Treg CD4^+^ T cells among splenocytes during persistent infection. These data reflect six to eight mice per time point representative of three independent experiments each with similar results. Bar, standard error.

The requirement for CD4^+^ T cells in bacterial clearance during persistent *Salmonella* infection may reflect contributions from either Foxp3-negative effector or Foxp3^+^ regulatory T cells (Tregs). To characterize the relative contributions of each CD4^+^ T cell subset during persistent infection, our initial studies enumerated the percent Foxp3^+^ cells among CD4^+^ T cells and the expansion kinetics of Foxp3^+^ and Foxp3-negative CD4^+^ T cells during persistent infection. Interestingly despite dramatic shifts in the percent and absolute number of CD4^+^ T cells among splenocytes, the percent Foxp3^+^ Tregs among CD4^+^ T cells remains remarkably stable and essentially unchanged at approximately 10% throughout the infection (Figure 4A and B). By extension, the absolute numbers of Foxp3^+^ Tregs and Foxp3-negative effector CD4^+^ T cells were also found to expand in parallel (Figure 4C). These findings suggest variations in the ratio of Foxp3^+^ Tregs among non-Treg effector CD4^+^ T cells alone does not account for the shift in relative T cell activation and change in infection tempo at early compared to late time points during persistent *Salmonella* infection.

### Dynamic shifts in Foxp3^+^ Treg suppressive potency

**Figure 5:**
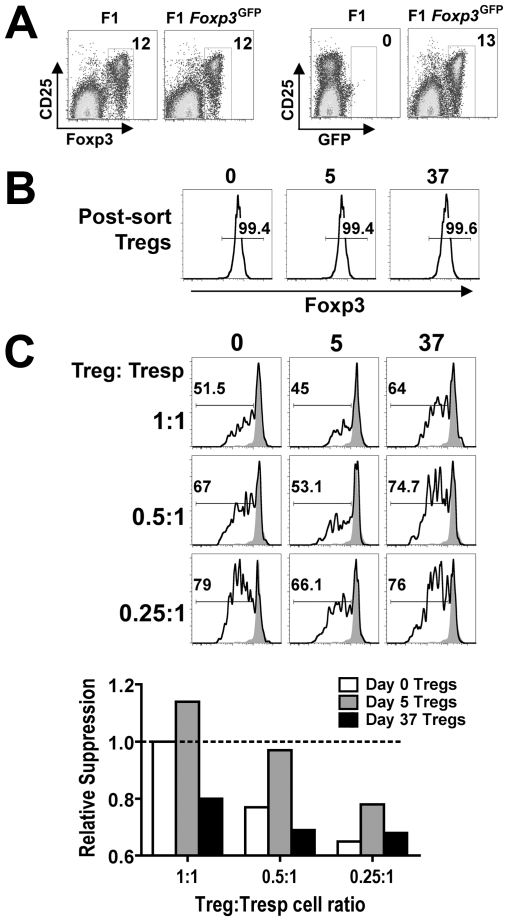
Dynamic regulation of Treg suppressive potency during persistent *Salmonella* infection. A. Percent Foxp3^+^ (left) and GFP^+^ (right) cells among CD4^+^ T cells from F1 and F1 *Foxp3* ^GFP/−^ mice. B. Expression of Foxp3^+^ after cell sorting for GFP^+^CD4^+^ cells from F1 *Foxp3* ^GFP/−^ mice at the indicated time points after infection. C. Percent CFSE^lo^ cells among CD45.1^+^CD4^+^ responder T cells (Tresp) after co-culture with the indicated ratio of GFP^+^(Foxp3^+^) Tregs isolated from mice at each time point after infection and stimulation with anti-CD3/CD28 (line histogram) or no stimulation (shaded histogram) (top). Relative suppression of CFSE dilution in CD45.1^+^CD4^+^ responder T cells by GFP^+^(Foxp3^+^) Tregs isolated at each time point after infection normalized to the suppression conferred by Tregs from naïve mice co-cultured with responder T cells at a 1∶1 ratio (dotted line) (bottom). These data are representative of three independent experiments each with similar results.

Since defined inflammatory cytokines and pathogen associated molecular patterns have each been shown to control Treg suppressive potency after stimulation *in vitro* [34], [35], [36], [37], [38], [39], we explored the possibility that intact pathogens and the ensuing immune response would also dictate shifts in Treg suppressive potency after infection *in vivo*. By extension, these shifts in relative Treg suppressive potency may impact the activation of non-Treg effector cells and overall tempo of persistent infection. Accordingly, we compared the suppressive potency for Foxp3^+^ Tregs isolated at early (day 5) and late (day 37) time points during persistent *Salmonella* infection. These specific time points where chosen because they reflect highly pronounced contrasts in T cell activation and directional changes in *Salmonella* bacterial burden, yet have comparable bacterial burdens (Figure 1 and 2). Nramp1-sufficient F1 *Foxp3* ^GFP^ reporter hemizygous male mice derived by intercrossing 129SvJ males with *Foxp3* ^GFP/GFP^ females (on the C57BL/6 background) that simultaneously allow persistent *Salmonella* infection and for all Tregs to be isolated based on cell sorting for GFP^+^(Foxp3^+^) cells were used in these experiments [23] (Figure 5A). By first enriching for CD4^+^ cells using negative selection, GFP^+^(Foxp3^+^) Tregs could be routinely isolated from naïve and *Salmonella*-infected F1 *Foxp3* ^GFP^ reporter mice each with ≥99% purity (Figure 5B). Potential differences in suppressive potency for GFP^+^(Foxp3^+^) Tregs isolated at each time point after infection were quantified by measuring their ability to inhibit the proliferation of responder CD4^+^ T cells isolated from naïve CD45.1 congenic mice after non-specific stimulation *in vitro* using previously defined methods [40], [41], [42].

Compared with Tregs isolated from F1 *Foxp3* ^GFP^ reporter mice prior to infection, the suppressive potency of Tregs isolated from mice day 5 after *Salmonella* infection was enhanced (Figure 5C). At the same Treg to responder T cell ratio, Foxp3^+^ Tregs from mice day 5 after infection consistently inhibited responder CD45.1^+^ T cell proliferation (CFSE dilution) more efficiently. These differences in suppression were eliminated when a 2-fold reduction in Treg to responder cell ratio from mice day 5 post-infection compared with undiluted Tregs from naïve mice were co-cultured with a fixed number of naïve responder cells (Figure 5C). In sharp contrast to increased suppression that occurs at this early post-infection time point, the suppressive potency for Tregs isolated from mice day 37 after infection was significantly reduced. Compared with Tregs isolated from mice 5 days after infection, the efficiency whereby Tregs isolated day 37 post-infection inhibited the proliferation of responder CD45.1^+^ T cells was reduced approximately 4-fold; and compared with Tregs isolated from naïve mice, their suppressive potency was reduced approximately 2-fold (Figure 5C). In other words, a 50% reduction in Treg to responder cell ratio for Tregs isolated from naïve mice, and a 75% reduction in ratio for Tregs from mice day 5 after infection each suppressed responder cell proliferation to the same extent as undiluted GFP^+^(Foxp3^+^) Tregs isolated from mice day 37 after infection. These results demonstrate that although the ratio of Foxp3^+^ Tregs and non-Treg effector CD4^+^ T cells remains unchanged, shifts in Treg suppressive potency that directly parallel the kinetics of T cell activation and infection tempo occur during the progression of persistent *Salmonella* infection.

**Figure 6:**
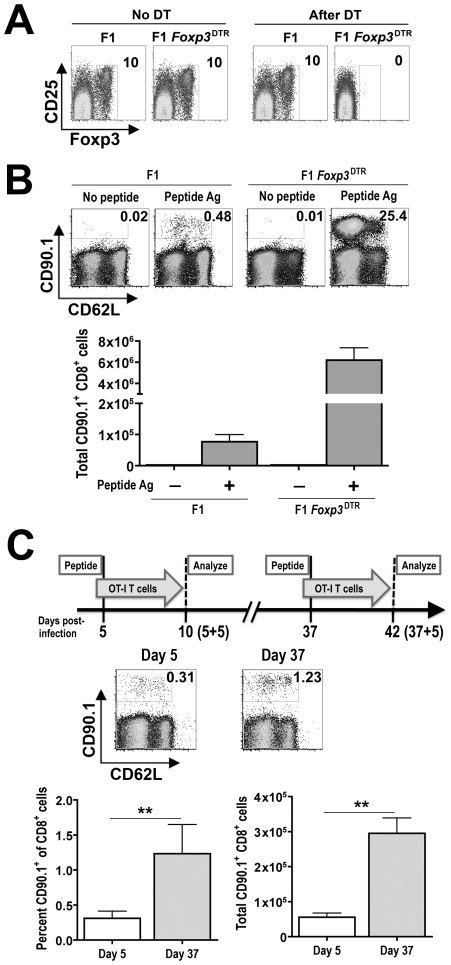
Shifts in Treg-mediated *in vivo* suppression during persistent *Salmonella* infection. A. Representative FACS plots demonstrating the efficiency whereby Foxp3^+^ Tregs are ablated with DT treatment in F1 *Foxp3* ^DTR^ compared with F1 *Foxp3* ^WT^ (F1) control mice. The numbers indicate the percent Foxp3-expressing among CD4^+^ T cells after DT treatment. B. Representative FACS plots demonstrating percent (top) CD90.1^+^ OT-1 T cells among CD8^+^ splenocytes and total number (bottom) of CD90.1^+^CD8^+^ splenocytes in Treg-sufficient (F1) or Treg-ablated (F1 *Foxp3* ^DTR^) mice day 5 after injection of OVA_257–264_ peptide or no peptide controls. C. Representative FACS plots (top) demonstrating percent CD90.1^+^ OT-I T cells among CD8^+^ splenocytes and composite data (bottom) depicting percent and total number CD90.1^+^CD8^+^ splenocytes after adoptive transfer into mice at the indicated time points after *Salmonella* infection and peptide stimulation. These data represent six to ten mice per group combined from three independent experiments each with similar results. Bar, standard error. **, *p*<0.01.

In complementary experiments, the relative suppressive environment dictated by Foxp3^+^ Tregs during *Salmonella* infection was further characterized. Specifically the expansion of adoptively transferred antigen-specific T cells after stimulation with cognate peptide at defined time points during persistent infection was enumerated. This approach exploits the use of F1 129SvJ X C57BL/6 mice as recipients for adoptively transferred T cells from TCR transgenic mice on the C57BL/6 background [43]. As a control to identify the overall contribution of Tregs in suppressing the expansion of adoptively transferred T cells *in vivo*, F1 *Foxp3* ^DTR^ hemizygous male mice derived from intercrossing 129SvJ males with *Foxp3* ^DTR/DTR^ female mice (on the C57BL/6 background), which allows targeted ablation of Foxp3^+^ Tregs by administering low-dose diphtheria toxin (DT) were used initially [24]. We found 1.0 µg (50 µg/kg) DT given on two consecutive days was sufficient for ≥99% ablation of Foxp3^+^ Tregs, and continued DT dosing (0.2 µg every other day) was able to maintain this level of Treg ablation in *Foxp3* ^DTR^ mice on the F1 background (Figure 6A). These results are consistent with the reported efficiency whereby Foxp3^+^ Tregs are selectively ablated in *Foxp3* ^DTR^ mice on the C57BL/6 background [24]. Although *in vivo* injection of cognate OVA_257–264_ peptide could stimulate only modest levels of expansion for adoptively transferred T cells from OT-1 TCR transgenic mice in Treg-sufficient mice, the expansion magnitude was increased >50-fold in Treg-ablated F1 *Foxp3* ^DTR^ mice (Figure 6B). Importantly, the expansion of these adoptively transferred T cells was antigen-dependent because very few cells could be recovered from either Treg-ablated or Treg-sufficient recipient mice without peptide stimulation. Thus, Tregs actively suppress the expansion of peptide stimulated antigen-specific T cells *in vivo*, and the relative expansion of these exogenous cells is a reflection of Treg suppressive potency.

Using this approach, the relative expansion of exogenous T cells from OT-1 TCR transgenic mice after adoptive transfer into *Salmonella* infected F1 129SvJ X C57BL/6 and stimulation with cognate OVA_257–264_ peptide was enumerated. The percent and total numbers of OT-1 T cells was increased 4-fold and 5-fold, respectively, after adoptive transfer into mice at late (day 37) compared with early (day 5) time points during persistent infection (Figure 6C). Thus, the *in vivo* environment at later compared with early time points during persistent *Salmonella* infection is significantly more permissive for peptide-stimulated T cell expansion. These results, together with the reductions in suppressive potency for GFP^+^(Foxp3^+^) cells isolated *ex vivo* from mice at early compared with late time points (Figure 5C), and the critical role for Foxp3^+^ Tregs in controlling exogenous T cell expansion in response to cognate peptide (Figure 6A and B) clearly illustrate reductions in Treg suppressive potency occur from early to late points during persistent *Salmonella* infection. Furthermore, given the sharp dichotomy in infection tempo at these specific time points, these results suggest enhanced Treg suppression early after infection restrains effector T cell activation that allows progressively increasing *Salmonella* bacterial burden, while diminished Treg suppression at later time points allows enhanced T cell activation that more efficiently controls the infection.

### Dynamic regulation of Treg-associated molecules that control suppression

**Figure 7:**
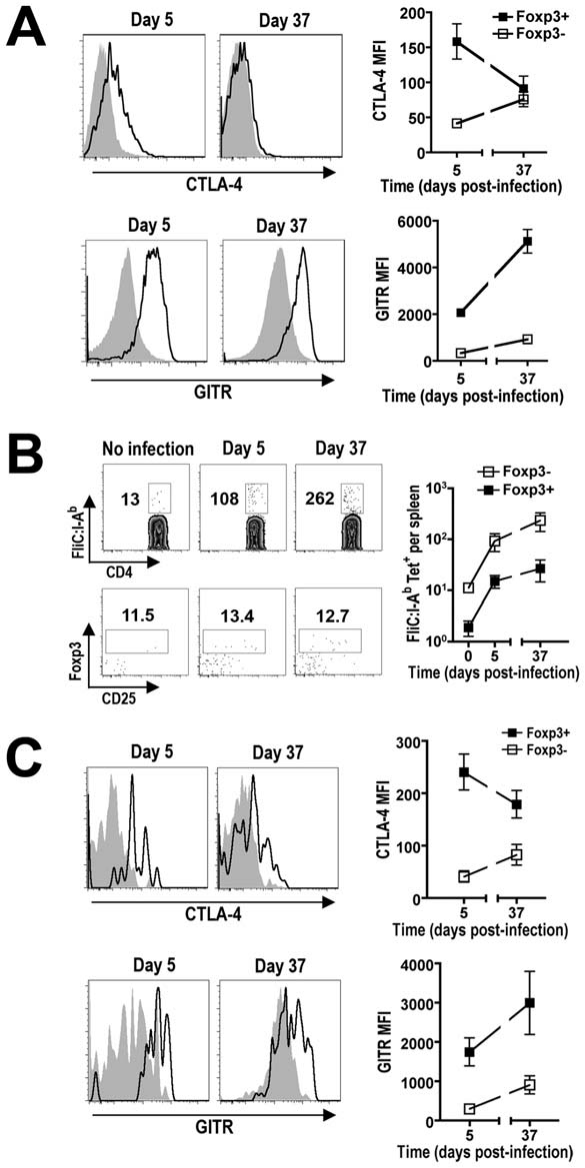
Expression of Treg-associated effector molecules during persistent *Salmonella* infection. A. The relative expression of CTLA-4 and GITR on Foxp3^+^ Tregs (line histogram) or Foxp3-negative CD4^+^ T cells (shaded histogram) at the indicated time points during persistent infection. B. Expansion of *Salmonella* FliC_431–439_-specific CD4^+^ T cells, and Foxp3-expression among these cells after staining with FliC:I-A^b^ tetramer and magnetic bead enrichment. The numbers in each plot represent the average cell number and percent Foxp3^+^ cells from 12 mice per time point combined from four independent experiments. C. Expression of CTLA-4 or GITR on FliC_431–439_-specific Foxp3^+^ Tregs (line histogram) and Foxp3-negative CD4^+^ T cells (shaded histogram) at early (day 5) and late (day 37) time points during persistent *Salmonella* infection. These data reflect six mice per time point representative of two independent experiments each with similar results. Bar, standard error.

Multiple Treg-associated cell surface and secreted molecules have been implicated to mediate immune suppression by these cells. For example, increased expression of CTLA-4, IL-10, Tgf-β, Granzyme B, ICOS, PD-1, and CD39 each have been shown independently to coincide with enhanced Treg suppressive potency [44], [45], [46], [47], [48], [49], [50], [51], [52], [53], while expression of other Treg cell-intrinsic molecules (e.g. GITR, OX40) each parallel reductions in suppressive potency [54], [55], [56]. Although the relative importance of each defined molecule varies significantly depending upon the experimental model used, the relative expression of Treg cell-intrinsic signals that either stimulate or inhibit suppression likely dictates the overall suppressive potency of Tregs. Therefore, we quantified the relative expression of each molecule on Foxp3^+^ Tregs to explore how the observed shifts in suppression potency from early to late time points during persistent *Salmonella* infection correlate with changes in their expression (Figure 7A and Figure S1). Consistent with the drastic reduction in suppressive potency, significant shifts in expression for some Treg-associated molecules between day 5 and day 37 post-infection were identified. For example, molecules that have independently been associated with diminished Treg suppression potency such as reduced CTLA-4 and increased GITR expression were found for Foxp3^+^ Tregs from mice day 5 compared with day 37 after infection [46], [54], [55] (Figure 7A). By contrast, more modest or minimal changes were found for other Treg-associated molecules implicated to mediate suppression (e.g. CD39, IL-10, Granzyme B, PD-1, and Tgf-β) [47], [48], [49], [50], [51], [52], [53], [56] (Figure S1). Thus, reduction in Treg suppressive potency during the progression of persistent *Salmonella* infection directly parallels reduced CTLA-4 and increased GITR expression that each independently correlates with this shift in suppression.

### *Salmonella* FliC-specific CD4^+^ T cells expand during persistent infection

Given the importance of pathogen-specific Tregs in controlling pathogen-specific effector cells in other models of persistent infection [57], [58], [59], the expansion kinetics and relative expression of Treg-associated effector molecules were also characterized for *Salmonella*-specific Tregs. The best characterized *Salmonella*-specific, I-A^b^-restricted MHC class II antigen is the flagellin FliC_431–439_ peptide [60]. Using tetramers with specificity for this antigen and magnetic bead enrichment, naïve C57BL/6 mice have been estimated to contain ∼20 FliC_431–439_-specific CD4^+^ T cells [61]. Using these same techniques, we find similar numbers of FliC_431–439_-specific CD4^+^ T cells in naïve F1 mice prior to *Salmonella* infection (Figure 7B). As predicted after *Salmonella* infection, the numbers of these FliC_431–439_-specific CD4^+^ T cells expand reaching ∼10-fold and 20-fold increased cell numbers day 5 and 37 post-infection, respectively (Figure 7B). Interestingly, for FliC_431–439_-specific CD4^+^ cells identified in this manner, ∼10% were Foxp3^+^ in F1 mice prior to and at each time point after infection (Figure 7B). Thus, FliC_431–439_-specific Tregs and effector T cells expand in parallel during this persistent infection, and these results are consistent with the stable percentage of Foxp3^+^ Tregs among bulk CD4^+^ T cells (Figure 4). Although the relatively small number (∼1–2 cells per mouse) of FliC_431–439_-specific Foxp3^+^ Tregs in naïve mice precluded further analysis beyond these absolute cell numbers, the expansion of FliC_431–439_-specific Tregs and non-Treg effector CD4^+^ T cells at early and late time points after infection allowed the relative expression of likely determinants of Treg suppression to be characterized. FliC_431–439_-specific Tregs were found to down-regulate CTLA-4 and up-regulate GITR expression, as infection progressed from early to late time points to a similar extent in FliC_431–439_-specific compared with bulk Tregs at these same time points after infection (Figure 7A and C). Thus, the relative expression of Treg-intrinsic molecules known to stimulate or impede immune suppression occurs for both pathogen-specific and bulk Foxp3^+^ Treg cells, and these changes directly coincide with reductions in their suppressive potency that occurs from early to late time points during persistent infection.

### Reduced impact of Treg-ablation from early to late time points during persistent infection

**Figure 8:**
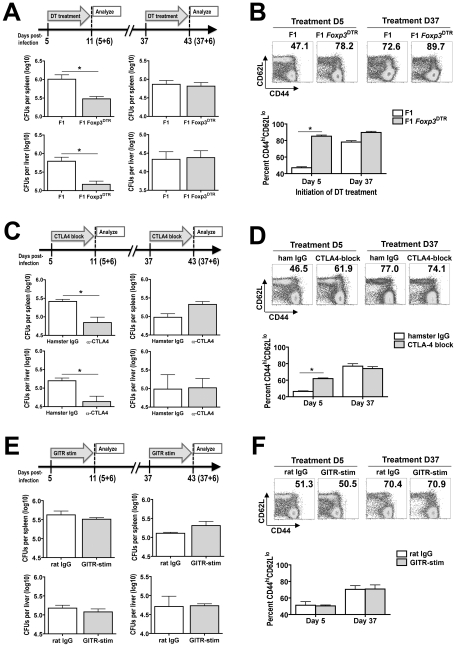
Relative impacts after Treg ablation on the tempo of persistent *Salmonella* infection. A. Number of recoverable *Salmonella* CFUs from spleen (top) and liver (bottom) in Treg-ablated F1 *Foxp3* ^DTR^ compared with Treg-sufficient F1 control mice when DT treatment was initiated at either early (day 5) or late (day 37) time points during persistent infection. B. Percent CD44^hi^CD62L^lo^ among CD4^+^ T cells in Treg-ablated F1 *Foxp3* ^DTR^ compared with Treg-sufficient F1 control mice when DT treatment was initiated on either day 5 or day 37 during persistent infection. C. Number of recoverable *Salmonella* CFUs from spleen (top) and liver (bottom) following CTLA-4 blockade beginning at either early (day 5) or late (day 37) time points post-infection in F1 mice. D. Percent CD44^hi^CD62L^lo^ among CD4^+^ T cells when CTLA-4 blockade was initiated at either early (day 5) or late (day 37) time points post-infection. E. Number of recoverable *Salmonella* CFUs from spleen (top) and liver (bottom) following treatment with GITR-stimulating antibody beginning at either early (day 5) or late (day 37) time points post-infection in F1 mice. F. Percent CD44^hi^CD62L^lo^ among CD4^+^ T cells when GITR stimulation was initiated at either early (day 5) or late (day 37) time points post-infection. These data reflect six to ten mice per group combined from two to three independent experiments each with similar results. Bar, standard error. *, *p*<0.05.

To more definitively identify the relative importance of Treg-mediated immune suppression on the progression of persistent *Salmonella* infection, the impacts of Treg ablation on infection tempo and T cell activation were enumerated at early and late time points after infection. Given the striking contrasts in suppressive potency for Foxp3^+^ Tregs, directional changes in bacterial burden, and effector T cell activation between mice day 5 versus day 37 post-infection, the relative impact caused by Treg ablation using F1 *Foxp3* ^DTR^ mice (Figure 6A) on infection tempo beginning at these time points were enumerated. In agreement with their essential role in maintaining and sustaining peripheral tolerance [24], Treg-ablated mice began to appear lethargic and dehydrated beginning day 8 after the initiation of DT treatment in *Salmonella*-infected mice. Thus, the impacts of Treg ablation on infection tempo and T cell activation were limited to discrete 7-day windows during persistent *Salmonella* infection. For mice that received DT beginning day 5 post-infection, significantly reduced numbers of recoverable *Salmonella* CFUs were found for Treg-ablated F1 *Foxp3* ^DTR^ compared with Treg-sufficient F1 *Foxp3* ^WT^ control mice 6 days after the initiation of DT treatment (day 5+6) (Figure 8A). These reductions in bacterial burden with Treg ablation early after infection were paralleled by significantly increased T cell activation (percent CD44^hi^CD62^lo^ T cells) (Figure 8B). Importantly, the reductions in *Salmonella* bacterial burden in Treg-ablated mice cannot be attributed to non-specific effects related to DT treatment because both Treg-ablated F1 *Foxp3* ^DTR^ and Treg-sufficient F1 *Foxp3* ^WT^ control mice each received identical doses of this reagent, nor could they be attributed to cell death-induced inflammation triggered by dying Tregs because no significant reductions in recoverable CFUs were found for F1 *Foxp3* ^DTR/WT^ heterozygous female mice where ∼50% Tregs express the high affinity DT receptor and are eliminated following DT treatment (Figure S2). By contrast, Treg ablation beginning later after infection (day 37) when T cells are already highly activated caused no significant change in *Salmonella* bacterial burden and only a modest incremental increase in T cell activation between Treg-ablated F1 *Foxp3* ^DTR^ compared with Treg-sufficient F1 *Foxp3* ^WT^ control mice (Figure 8A and B). Thus, the relative impact of Treg ablation at early and late time points on infection outcome directly parallel the differences in their suppressive potency. Together, these results demonstrate enhanced Treg suppressive potency at early infection time points restrains effector T cell activation and allows progressively increasing bacterial burden. By extension, Treg ablation at these early time points markedly increases T cell activation and significantly reduces the bacterial burden (Figure 8A and B). Reciprocally, at later time points after infection when Treg suppressive potency is diminished, the relative contribution of Foxp3^+^ Tregs on T cell activation and bacterial clearance is reduced (Figure 8A and B). Thus, dynamic regulation of Treg suppression dictates the balance between pathogen proliferation and clearance during the course of persistent *Salmonella* infection.

Given the drastic shifts in Treg-associated expression of CTLA-4 and GITR that each correlates with the reduced suppressive potency of these cells from early to late time points during persistent *Salmonella* infection, additional experiments sought to identify the relative importance of these molecules in dictating infection tempo using well characterized CTLA-4 blocking (clone UC10-4F10) or GITR-stimulating (clone DTA-1) monoclonal antibodies [54], [62], [63], [64]. Consistent with the essential role for CTLA-4 in Treg suppression during non-infection conditions *in vivo* [46], significant reductions in *Salmonella* recoverable CFUs and accelerated T cell activation were found with CTLA-4 blockade initiated beginning day 5 after *Salmonella* infection, and the magnitude of these changes paralleled those following DT-induced Treg ablation in F1 *Foxp3* ^DTR^ mice (Figure 8C and D). Since Foxp3-negative cells also express CTLA-4, albeit at significantly reduced levels compared with Foxp3^+^ Tregs (Figure 7), we further explored the relative contribution of CTLA-4 blockade in the absence of Foxp3^+^ Tregs. Consistent with the reduced levels of CTLA-4 expression on Foxp3-negative CD4^+^ T cells, the effects of CTLA-4 blockade were eliminated with Foxp3^+^ Treg ablation (Figure S3). By extension, at later time points after infection (day 37) when CTLA-4 expression is down-regulated on Foxp3^+^ Tregs, no significant change in *Salmonella* bacterial burden or T cell activation occurred with CTLA-4 blockade (Figure 8C and D). By contrast to these results with CTLA-4 blockade that directly recapitulates the effects of Treg ablation at early and late time points during persistent infection, treatment with a monoclonal antibody that stimulates cells through GITR caused no significant changes in *Salmonella* bacterial burden or T cell activation when initiated at either early or late time points during persistent infection (Figure 8E and F). Together, these results suggest the dynamic regulation of Treg suppressive potency during *Salmonella* infection is predominantly mediated by shifts in CTLA-4 expression, and reduced CTLA-4 expression by Tregs during the progression of this persistent infection dictates reduced suppression with enhanced effector T cell activation and bacterial clearance.

## Discussion

The balance between immune activation required for host defense, and immune suppression that limits immune-mediated host injury is stringently regulated during persistent infection [6], [7]. Although Tregs have been widely implicated to control the activation of immune host defense components during infection, their role in dictating the natural progression of persistent infection remains undefined. In this study, we report two distinct phases of effector T cell activation with opposing directional changes in pathogen burden in a mouse model of persistent *Salmonella* infection. Delayed T cell activation associated with increasing bacterial burden occurs early, while enhanced T cell activation that parallels reductions in pathogen burden occurs later during infection. Remarkably, significant reductions in Treg suppressive potency between early and late infection time points directly coincide with these differences in infection tempo. In complementary experiments, the significance of these shifts in Treg suppressive potency were verified by directly enumerating the relative impact of Treg ablation on infection tempo at early and late infection time points. Together, these results demonstrate dynamic changes in Foxp3^+^ Treg suppressive potency dictate the natural course and progression of this persistent infection.

Along with two recent studies characterizing infection outcome with Foxp3^+^ Treg ablation after mucosal HSV-2, systemic LCMV, and footpad West Nile virus infections [25], [26], these are the first studies to characterize the importance of Tregs during infection using *Foxp3* ^DTR^ transgenic mice. These results comparing infection outcome after Treg manipulation based on their lineage-defining marker, Foxp3, allow the importance of Tregs to be more precisely characterized compared with other methods that identify and manipulate Tregs using surrogate markers (e.g. CD25 expression) that are not expressed exclusively by these cells. Interestingly, while Treg ablation caused increased pathogen burden, delayed arrival of acute inflammatory cells, and accelerated mortality after HSV-2, LCMV, or West Nile virus infections [25], [26], we find contrasting reductions in pathogen burden and increased T cell activation with Treg ablation at early, but not late time points during persistent *Salmonella* infection. However, the reductions in *Salmonella* pathogen burden with early Treg ablation are consistent with reduced *Mycobacterium tuberculosis* pathogen burden after partial Treg depletion using bone marrow chimera mice reconstituted with mixed cells containing congenically-marked Foxp3^+^ Tregs and Foxp3-deficient cells [65]. Together, these studies comparing infection outcome after Treg ablation using Foxp3-specific reagents highlight interesting and divergent functional roles for Foxp3^+^ Tregs during specific infections. The reasons that account for these differences – whether they are related to differences between bacterial versus viral pathogens or between pathogens that primarily cause acute versus persistent infection, are important areas for additional investigation, and require the characterization of infection outcomes after Treg manipulation using Foxp3-specific reagents with other pathogens.

The dynamic regulation of Treg suppressive potency during *Salmonella* infection we demonstrate here is consistent with the ability of inflammatory cytokines and purified Toll-like receptor (TLR) ligands to each control Treg suppression after stimulation *in vitro* [34], [35], [36], [37], [38], [39]. However, since these stimulation signals in isolation trigger opposing directional changes in suppressive potency, the specific contribution for each on changes in Treg suppression during infection is unclear. Therefore, the cumulative impact of multiple TLR ligands expressed by intact pathogens and the ensuing immune response on changes in Treg suppression is best characterized for Tregs isolated directly *ex vivo* after infection. The increased suppressive potency for Foxp3^+^ Treg at early time points after *Salmonella* infection we demonstrate here is consistent with the increased suppressive potency for CD25^+^CD4^+^ cells isolated day 5 after *Plasmodium yoelii* and day 10 after HSV-1 infection, as well as CD25^+^CD4^+^ cells isolated in the acute (day 12) and chronic phase (day 28) after *Heligmosomoides polygyrus* infection [14], [66], [67]. However, our results build upon and extend the significance of these findings in three important respects. First, by isolating Tregs based on Foxp3 rather than CD25 expression, the limitations imposed by contaminating non-Treg CD25^+^ effector T cells in subsequent *ex vivo* functional analysis is bypassed.

Secondly, although an increase in CD25^+^CD4^+^ T cell suppression early after infection when pathogen proliferation occurs, potential shifts in Treg suppression at later time points during the natural progression of persistent infections has not been previously demonstrated. In this regard, the relatively short time interval that separates pathogen proliferation and clearance during persistent *Salmonella* infection is ideally suited for comparing differences in relative importance and suppressive potency for Tregs during these contrasting stages of infection. Using this model, we demonstrate significant reductions in Treg suppressive potency between early and late time points after infection that enables robust immune cell activation required for pathogen clearance. Despite these changes in suppressive potency, the percentage of Tregs among bulk and *Salmonella* FliC-specific CD4^+^ T cells each remained relatively constant throughout infection. These findings are consistent with the stable ratio of Tregs to effector CD4^+^ T cells during other models of persistent infection, and represent a striking contrast to the selective priming and expansion of pathogen-specific Foxp3-negative CD4^+^ T cells that occurs after acute *Listeria monocytogenes* infection [58], [65], [68], [69]. Thus, the priming and expansion of pathogen-specific Tregs may be an important distinguishing feature between pathogens that cause acute rather than persistent infection.

The development and refinement of methods for MHC class II tetramer staining and magnetic bead enrichment has allowed the precise identification of very small numbers of T cells with defined specificity from naïve mice [61]. Using these techniques, we find in this and a recent study [69] that Foxp3^+^ Tregs comprise approximately 10% of CD4^+^ T cells with specificity to both the FliC_431–439_ and 2W1S_52–68_ peptide antigens, respectively. Together, these results suggest previously under-appreciated overlap in the repertoire of antigens recognized by Foxp3^+^ Tregs compared with non-Treg CD4^+^ effectors in naïve mice [70], [71], [72]. However, more considerable overlap in the specificity of these two cell types is consistent with the TCR repertoires of human peripheral Tregs and non-Tregs based on genomic analysis of TCR sequences [73], [74]. Thus, additional studies that examine the percent Tregs among CD4^+^ T cells with other defined antigen-specificities using recently developed tetramer-based enrichment techniques are warranted.

Lastly, by enumerating the relative expression of defined Treg-associated molecules that have been implicated to directly mediate or inhibit suppression, the complexity whereby Tregs maintain the balance between immune activation and suppression becomes more clearly defined. For example, shifts in suppressive potency for Tregs isolated from early compared to late time points during persistent infection are paralleled by significant changes in the expression of numerous Treg cell-intrinsic molecules that have been demonstrated in other experimental models to control and/or mediate suppression [44], [45] (Figure 7 and Figure S1). In particular, the drastic reductions in suppressive potency that occurs for Tregs isolated from mice day 5 compared with day 37 after infection is associated with significant reductions in CTLA-4 expression and increased expression of GITR on both bulk Foxp3^+^ Tregs and *Salmonella* FliC_431–439_-specific Foxp3^+^ Tregs (Figure 7). Based on these results, the relative contributions of CTLA-4 and GITR in controlling suppression by Foxp3^+^ Tregs during persistent infection were investigated using antibody reagents that block CTLA-4 or stimulate cells through GITR. We find that CTLA-4 blockade alone is sufficient to recapitulate the effects of Treg ablation on *Salmonella* infection tempo, while GITR stimulation had no significant effect (Figure 8). These results are consistent with the recent demonstration that CTLA-4 expression on Foxp3^+^ Tregs is essential for maintaining peripheral tolerance [46], [75]. Our results expand upon these findings by demonstrating the importance of dynamic CTLA-4 expression on Tregs during persistent infection that controls the kinetics of effector T cell activation and overall infection tempo.

The increase in Treg suppressive potency at early time points after *Salmonella* infection is consistent and may provide the mechanistic basis that explains the relative immune suppression previously observed during this infection [76], [77], [78], [79], [80]. Interestingly, increased Treg suppressive potency early after infection has also been described after viral and parasitic pathogens [14], [66], [67]. Is enhanced Treg suppression early after infection advantageous for the host, the pathogen, or both? Our ongoing studies are aimed at identifying the signals activated during *Salmonella* infection that trigger these changes, and Treg-intrinsic molecules that sense and dictate this augmentation in suppressive potency. Perhaps more intriguing are the molecular signals during natural infection that trigger reductions in Treg suppression that transform blunted immune effectors early after infection into more potent mediators of pathogen clearance. Given the multiple known pathogen-associated molecular patterns expressed by *Salmonella* (e.g. LPS, flagellin, porins, and CpG DNA) that each stimulate immune cells through defined Toll-like and other pattern recognition receptors [81], [82], [83], [84], [85], [86], together with the enormous potential for cell intrinsic TLR-stimulation on Tregs to alter their suppressive potency [34], [35], [36], [38], [87], it is tempting to hypothesize that shifts in the expression of individual, multiple, or cumulative TLR ligands during persistent infection controls the relative expression of Treg-associated molecules that mediate suppression. In this regard, our ongoing studies are also aimed at identifying the *Salmonella*-specific ligands and their corresponding host receptors that dictate these reductions in Treg suppression during the progression of this persistent infection. We believe these represent important prerequisites for developing new therapeutic intervention strategies aimed at accelerating the transition to pathogen clearance and further unraveling the pathogenesis of typhoid fever caused by *Salmonella* infection.

## Materials and Methods

### Ethics statement

These experiments were conducted under University of Minnesota IACUC approved protocols (0705A08702 and 1004A80134) entitled “Regulatory T cells dictate immunity during persistent *Salmonella* infection”. The guidelines followed for use of vertebrate animals were also created by the University of Minnesota IACUC.

### Mice

129SvJ males and C57BL/6 females were purchased from the National Cancer Institute. F1 mice were generated by intercrossing 129SvJ males with C57BL/6 females. F1 *Foxp3* ^GFP/−^ and F1 *Foxp3* ^DTR/−^ hemizygous males and F1 *Foxp3* ^DTR/WT^ heterozygous females were derived by intercrossing 129SvJ males with *Foxp3* ^GFP/GFP^ or *Foxp3* ^DTR/DTR^ females, respectively [23], [24]. Both *Foxp3* ^GFP/GFP^ and *Foxp3* ^DTR/DTR^ females have been backcrossed to C57BL/6 mice for over 15 generations. OT-1 TCR transgenic mice that contain T cells specific for the OVA_257–264_ peptide were maintained on a RAG-deficient CD90.1^+^ background. All mice were used between 6–8 weeks of age and maintained within specific pathogen-free facilities.

### Bacteria

The virulent *Salmonella enterica* serotype Typhimurium strain SL1344 has been described [29], [88]. For infections, SL1344 was grown to log phase in brain heart infusion media at 37°C, washed and diluted with saline to a final concentration of 1×10^4^ CFUs (for infection in F1 mice) or 1×10^2^ CFUs (for infection in C57BL/6 mice) per 200 µL, and injected intravenously through the lateral tail vein. At the indicated time points after infection, mice were euthanized and the number of recoverable *Salmonella* CFUs enumerated by plating serial dilutions of the spleen and liver organ homogenate onto agar plates.

### Reagents

Antibodies and other reagents for cell surface, intracellular, or intranuclear staining were purchased from BD Biosciences (San Jose, CA) or eBioscience (San Diego, CA), and used according to the manufacturers' recommendations. For measuring cytokine production by T cells, splenocytes were stimulated *ex vivo* with anti-mouse CD3 and anti-mouse CD28 (each at 5 µg/mL) in the presence of brefeldin A for 5 hours prior to intracellular cytokine staining. Antibodies used for depletion, blocking or stimulation experiments were purchased from BioXcell (West Lebanon, NH). For T cell depletions, purified anti-mouse CD4 (clone GK1.5) and anti-mouse CD8 (clone 2.43) antibodies were diluted to a final concentration of 750 µg per 1 mL in sterile saline and injected intraperitoneally on days 31 and 34 post-infection. Additional injections were given on days 38 and 41 post-infection in experiments where depletion was maintained up to 14 days. For CTLA-4 blockade and GITR stimulation, anti-mouse CTLA4 (clone UC10-4F10), anti-mouse GITR (clone DTA-1), or isotype control antibodies (hamster IgG or rat IgG, respectively) were diluted to a final concentration of 500 µg per 1 mL in sterile saline and injected intraperitoneally beginning either day 5 or day 37 post-infection followed by an additional injection of 250 µg of the same antibody three days later [54], [62], [63], [64]. For Foxp3^+^ Treg ablation, purified diphtheria toxin (DT; Sigma-Aldrich, St. Louis, MO) was dissolved in saline and administered intraperitoneally to F1 *Foxp3* ^WT^ control, F1 *Foxp3* ^DTR/−^, or F1 *Foxp*3^DTR/WT^ mice at 50 µg/kg body weight for two consecutive days beginning at indicated time point after infection, and then maintained on a reduced dose of DT thereafter (10 µg/kg body weight every other day).

### In vitro and in vivo suppression assays

For enumerating relative Treg suppression *in vitro*, Foxp3^+^GFP^+^ Tregs were isolated from F1 *Foxp3* ^GFP/−^ mice by enriching CD4^+^ splenocytes first with negative selection using magnetic bead cell isolation kits (Miltenyi Biotec, Auburn, CA). GFP^+^(Foxp3^+^) Tregs were further purified by staining and sorting for CD4^+^GFP^+^ cells using a FACSAria cell sorter. The purity of CD4^+^Foxp3^+^ cells post-sort was verified to be >99%. Responder CD4^+^ T cells were isolated from naïve CD45.1^+^ mice, CFSE-labeled under standard conditions (5 µM for 10 min), and co-cultured in 96-well round bottom plates (2×10^4^ cells/100 µL) at the indicated ratio of purified GFP^+^(Foxp3^+^) Tregs and responder CD45.1^+^CD4^+^ T cells. The relative suppressive potency for Tregs was enumerated by comparing the proliferation (CFSE dilution) in responder cells after co-culture and stimulation with anti-mouse CD3 and anti-mouse CD28 antibodies (1 µg/mL each) for 4 days. For enumerating relative Treg suppression *in vivo*, 2×10^4^ T cells from OT-1 TCR transgenic mice on a RAG CD90.1^+^ background were diluted in 200 µL sterile saline and injected intravenously at the indicated time points relative to Treg ablation or *Salmonella* infection followed by intravenous injection of purified OVA_257–264_ peptide (400 µg) the following day. For each experiment, the degree of OT-1 T cell expansion was enumerated five days later.

### MHC tetramer staining and enrichment

MHC class II tetramer staining and enrichment were performed as described [61], [69]. Briefly, splenocytes were harvested at the indicated time points after infection and incubated with 5–25 nM PE or APC-conjugated FliC_431–439_-specific tetramer in Fc block for 1 hour at room temperature. These cells were then incubated with anti-PE or anti-APC magnetic beads (Miltenyi Biotec, Auburn, CA) for 30 minutes on ice and column purified according to the manufacturer's instructions. The bound and unbound fractions were stained with fluorochrome-labeled antibodies for cell surface and intracellular staining. The absence of I-A^b^ FliC_431–439_ tetramer staining on CD8^+^ T cells, and among CD4^+^ T cells in the unbound fraction of cells after bead enrichment were used as independent markers to verify the specificity of tetramer staining using methods described (data not shown) [61].

### Statistics

The differences in number of recoverable bacterial CFUs, and the number and percent T cells among from different groups of mice were evaluated using the Student's *t* test (GraphPad, Prism Software) with *p*<0.05 taken as statistical significance.

## Supporting Information

Figure S1 Expression of Treg-associated molecules during persistent *Salmonella* infection. The relative expression of defined Treg cell-intrinsic molecules known to either enhance (CD39, Granzyme B, ICOS, IL-10, PD-1, Tgf-β) or impede (OX40) suppression on Foxp3^+^ Tregs (line histogram) or Foxp3-negative CD4^+^ T cells (shaded histogram) at the indicated time points during persistent infection. These data reflect six mice per time point representative of two independent experiments each with similar results. Bar, standard error.
(0.18 MB DOC)

Figure S2 Relative impact of Treg ablation on recoverable *Salmonella* CFUs in F1 control, Foxp3^DTR/−^ and Foxp3^DTR/WT^ mice. Number of recoverable *Salmonella* CFUs from the spleen (top) and liver (bottom) in Treg-ablated F1 Foxp3^DTR/−^ and partially Treg-ablated F1 Foxp3^DTR/WT^ mice compared with Treg-sufficient F1 control mice six days after the initiation of DT treatment beginning day 5 post-infection. These data reflect eight to twelve mice per group combined from three independent experiments each with similar results. Bar, standard error. *, *p*<0.05.
(0.08 MB DOC)

Figure S3 Foxp3^+^ Treg ablation eliminates the effects of CTLA-4 blockade early after *Salmonella* infection. Number of recoverable *Salmonella* CFUs (left) from spleen and liver following CTLA-4 blocking (α-CTLA-4) or isotype control (hamster IgG) antibody, and DT treatment in F1 Foxp3^DTR^ mice each beginning day 5 post-infection, and harvested six days thereafter (day 11 post-infection). Percent CD44^hi^CD62L^lo^ among CD4^+^ T cells (right) for each group of *Salmonella* infected mice. Bar, standard error.
(0.13 MB DOC)
