# Supplementary material for: Regulatory T Cell Suppressive Potency Dictates the Balance between Bacterial Proliferation and Clearance during Persistent Salmonella Infection
Source: PLoS Pathog. 2010 Aug 12;6(8):e1001043. doi: 10.1371/journal.ppat.1001043 (PMC2920851; doi:10.1371/journal.ppat.1001043)
Supplement: Figure S1 — Expression of Treg-associated molecules during persistent Salmonella infection. The relative expression of defined Treg cell-intrinsic molecules known to either enhance (CD39, Granzyme B, ICOS, IL-10, PD-1, Tgf-β) or impede (OX40) suppression on Foxp3+ Tregs (line histogram) or Foxp3-negative CD4+ T cells (shaded histogram) at the indicated time points during persistent infection. These data reflect six mice per time point representative of two independent experiments each with similar results. Bar, standard error. (0.18 MB DOC) [file ppat.1001043.s001.doc]

**­**
