# Supplementary material for: Regulatory T Cell Suppressive Potency Dictates the Balance between Bacterial Proliferation and Clearance during Persistent Salmonella Infection
Source: PLoS Pathog. 2010 Aug 12;6(8):e1001043. doi: 10.1371/journal.ppat.1001043 (PMC2920851; doi:10.1371/journal.ppat.1001043)
Supplement: Supplementary file 2 [file ppat.1001043.s002.doc]

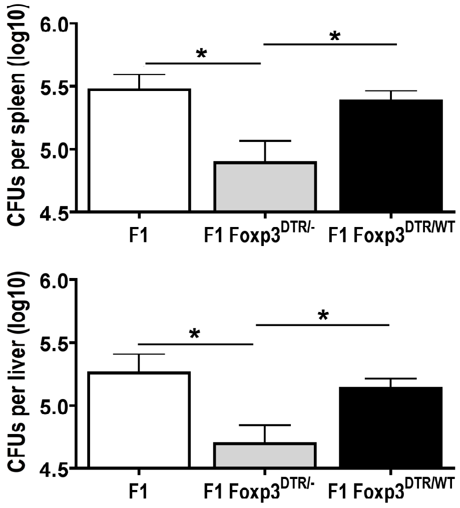

CFUs per spleen (log10)
6.0
5.5
5.0
4.5
*
*
F1
F1 Foxp3DTR/-
F1 Foxp3DTR/WT
CFUs per liver (log10)
6.0
5.5
5.0
4.5
*
*
F1
F1 Foxp3DTR/-
F1 Foxp3DTR/WT
