# Supplementary material for: Regulatory T Cell Suppressive Potency Dictates the Balance between Bacterial Proliferation and Clearance during Persistent Salmonella Infection
Source: PLoS Pathog. 2010 Aug 12;6(8):e1001043. doi: 10.1371/journal.ppat.1001043 (PMC2920851; doi:10.1371/journal.ppat.1001043)
Supplement: Supplementary file 3 [file ppat.1001043.s003.doc]

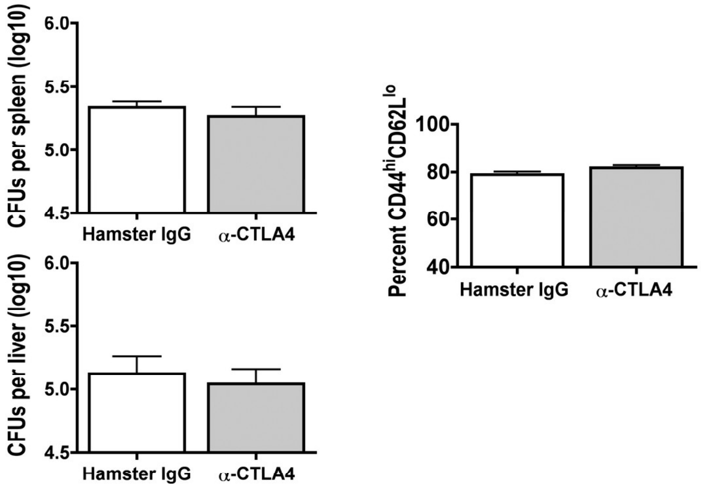
CFUs per spleen (log10)
6.0
5.5
5.0
4.5
Hamster IgG
α-CTLA4
CFUs per liver (log10)
6.0
5.5
5.0
4.5
Hamster IgG
α-CTLA4
Percent CD44hiCD62Llo
100
80
60
40
Hamster IgG
α-CTLA4
